# Functional and clinical characteristics of focal adhesion kinases in cancer progression

**DOI:** 10.3389/fcell.2022.1040311

**Published:** 2022-11-02

**Authors:** Zhaoyu Zhang, Jinlong Li, Simin Jiao, Guangda Han, Jiaming Zhu, Tianzhou Liu

**Affiliations:** ^1^ Department of Gastrointestinal Nutrition and Hernia Surgery, The Second Hospital of Jilin University, Changchun, Jilin, China; ^2^ Department of Surgical Oncology and General Surgery, The First Affiliated Hospital of China Medical University, Shenyang, Liaoning, China

**Keywords:** FAK, cancer progression, clinical significance, molecular mechanisms, prognosis

## Abstract

Focal adhesion kinase (FAK) is a non-receptor tyrosine kinase and an adaptor protein that primarily regulates adhesion signaling and cell migration. FAK promotes cell survival in response to stress. Increasing evidence has shown that at the pathological level, FAK is highly expressed in multiple tumors in several systems (including lung, liver, gastric, and colorectal cancers) and correlates with tumor aggressiveness and patient prognosis. At the molecular level, FAK promotes tumor progression mainly by altering survival signals, invasive capacity, epithelial-mesenchymal transition, the tumor microenvironment, the Warburg effect, and stemness of tumor cells. Many effective drugs have been developed based on the comprehensive role of FAK in tumor cells. In addition, its potential as a tumor marker cannot be ignored. Here, we discuss the pathological and pre-clinical evidence of the role of FAK in cancer development; we hope that these findings will assist in FAK-based clinical studies.

## 1 Introduction

Focal adhesion kinase (FAK) is a multifunctional tyrosine kinase protein encoded by *PTK2* (or *FAK*) that is overexpressed in tumor cells associated with adverse clinical outcomes (Zhou et al., 2018). As a non-receptor cytoplasmic tyrosine kinase and scaffolding protein located in the adhesive plaque, FAK mediates and integrates signals initiated by growth factor, integrin, vascular endothelial growth factor receptor (VEGFR), and G protein-coupled receptor. This activates downstream signals (such as PI3K, Akt, and MAPK) and regulates intracellular functions (Devaud et al., 2019; Fan et al., 2019). Moreover, growing evidence has revealed that FAK is involved in the regulation of multiple tumorigenic processes, including growth factor signaling, cell cycle progression, cell survival, migration, metastasis, angiogenesis, and the establishment of an immunosuppressive tumor microenvironment (TME) through kinase-dependent and independent scaffolding functions in the cytosol and nucleus (Haskell et al., 2003; Kobayashi et al., 2009; Osipov et al., 2019).

## 2 Characteristics of the focal adhesion kinase molecule

**FIGURE 1:**
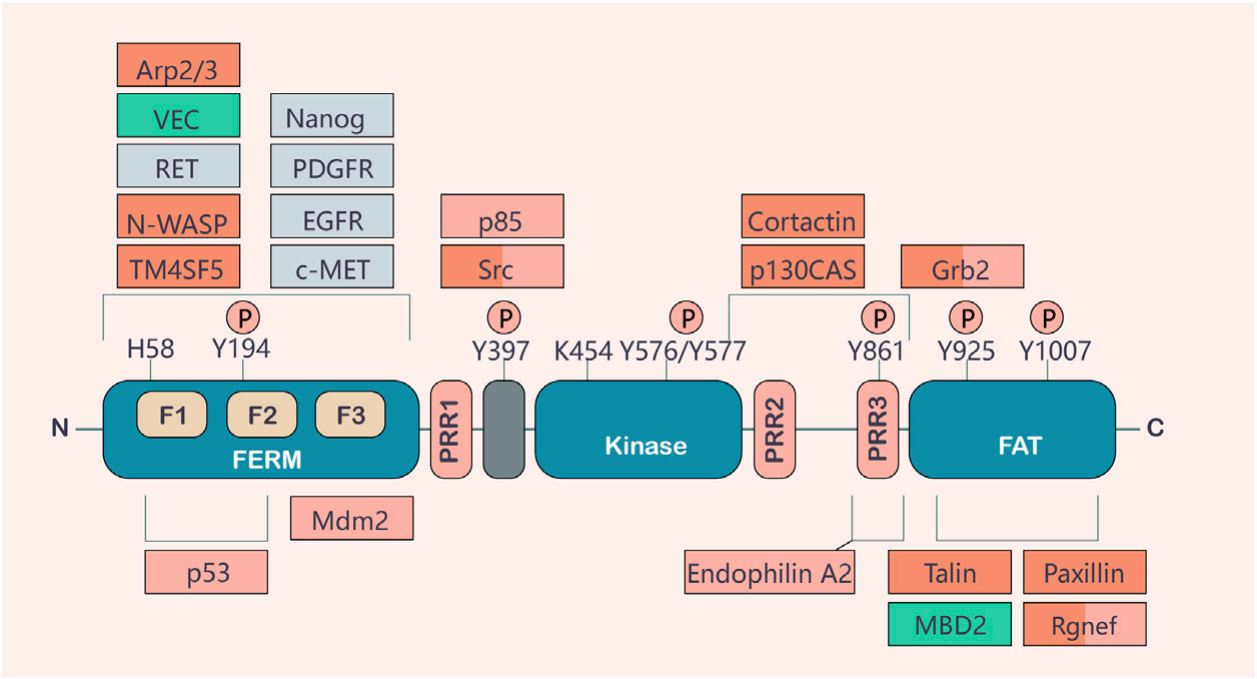
Basic structure and function of FAK. FAK consists of a central activation region and a protein band [4.1-Ezrin-Radioxin-moesin (FERM)] homologous structural domain located at the amino terminus and a carboxy-terminal focal adhesion targeting (FAT) structural domain. These two terminal structural domains are separated from the activation domain by a linker region containing proline-rich regions (PRRs). Important tyrosine phosphorylation (P) sites include Y397, K454, and H58, which play key roles in FAK activation. FAK binding partners are shown at their sites of interaction in FAK. The binding of different partners affects functions, such as cell motility (red), cell survival (orange), or both (red/orange). Actors involved in FAK activation are shown in gray, and important contributions to the tumor environment are shown in green (Sulzmaier et al., 2014).

The human gene encoding *FAK* (also known as *PTK2*) is located on chromosome 8q24.3, a region shown to have frequent aberrations in human oncology (Pylayeva et al., 2009; Schaller, 2010). The coding sequence of FAK, a highly conserved 125 kDa non-receptor tyrosine kinase, contains 34 exons (Corsi et al., 2006). FAK consists of an amino-terminal region containing a 4.1-Ezrin-Radixin-moesin (FERM) structural domain, a central kinase structural domain, and a carboxy-terminal focal adhesion targeting (FAT) structural domain (Alanko and Ivaska, 2016). Three proline-rich regions (PRRs) are anchored to the linkage region between these structural domains. Phosphorylation occurs at several important tyrosine residues, including the autophosphorylation site Tyr397, Tyr576/577 in the activation loop of the kinase structural domain, and Tyr861, Tyr925, and Tyr1007 in the C-terminal structural domain (Wu et al., 2022). It is well known that both the N- and C-terminal structural domains mediate the interaction of FAK with other proteins essential for activating its kinase structural domain and regulating different cellular functions. FAK is maintained in an inactive state through the binding of the FERM structural domain to the kinase structural domain, which prevents access to the critical autophosphorylation site tyrosine 397 (Tyr397) (Frame et al., 2010). After binding to the extracellular matrix or growth factors, integrins stimulate G protein-linked receptors, leading to a signaling substitution of the FERM structural domain. This results in Tyr397 autophosphorylation, conformational changes in FAK and/or its binding partners, and binding and/or regulation of downstream effector molecules (such as Src, MAPK, PI3K, paxlin, and Rac) (Frame et al., 2010). The C-terminal structural domain provides binding sites for proteins, such as p130Cas and VEGFR3 (Frame et al., 2010). It includes the FAT sequence, which is responsible for FAK localization to focal adhesions and facilitates its co-localization with integrins by interacting with integrin-related proteins. The lipid domain is also associated with several Rho GTPases, such as p190RhoGF (Aboubakar Nana et al., 2019a) (Figure 1).

## 3 Focal adhesion kinase regulates tumor development and progression

FAK expression and activation are regulated by several mechanisms: at the gene level by gene amplification (Agochiya et al., 1999; Okamoto et al., 2003); at the RNA level by selective splicing (Corsi et al., 2006; Devaud et al., 2019) or FAK mRNA upregulation (Tremblay et al., 1996; Fujii et al., 2004); at the translational and post-translational levels *via* phosphorylation (Imaizumi et al., 1997), dephosphorylation (Hauck et al., 2001); and non-coding RNA regulation (Egawa et al., 2016; Cheng et al., 2017; Qu et al., 2017; Wang et al., 2019; Yan et al., 2019; Pan and Xie, 2020; Tang et al., 2020; Zhang et al., 2021a). FAK plays an integral role in the development of various tumors through these mechanisms.

**FIGURE 2:**
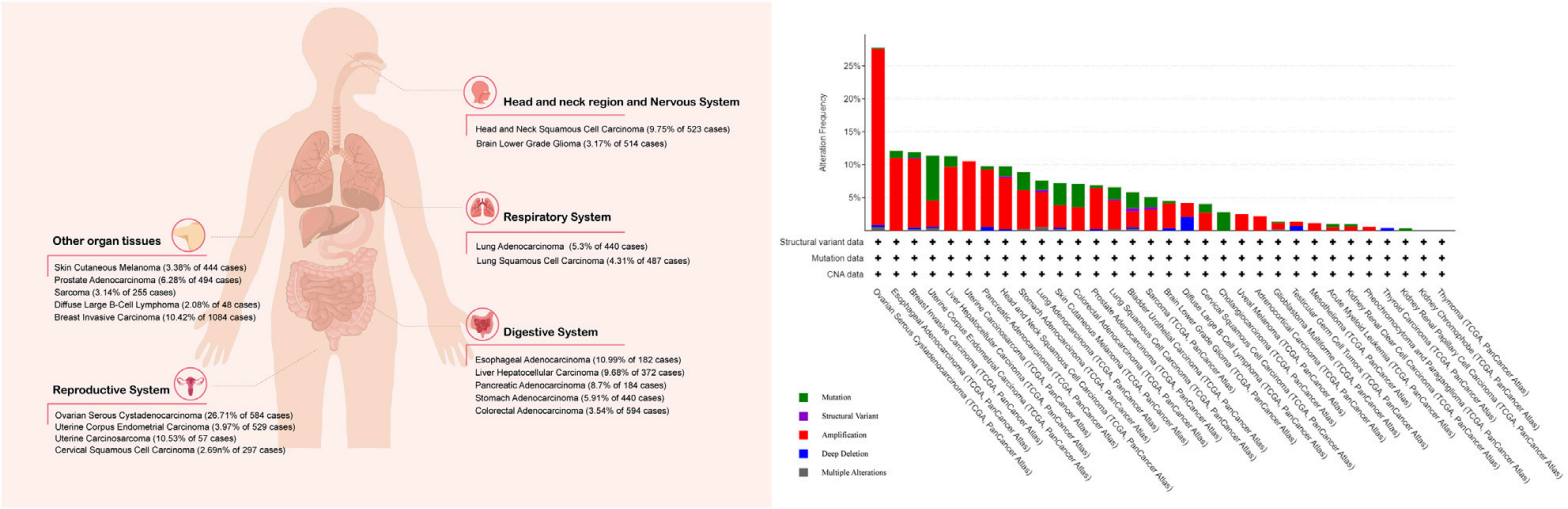
Percentage of tumor samples with increased FAK mRNA. The Cancer Genome Atlas was queried using the cBioPortal (cBioPortal for Cancer Genomics: PTK2 in Adrenocortical Carcinoma (TCGA, PanCancer Atlas) and 31 other studies). The search criteria included mRNA expression data (Z-scores for all genes) and tumor datasets with mRNA data.

Multiple methods, including western blotting (WB), quantitative real-time polymerase chain reaction (qPCR), and immunohistochemistry (IHC), have shown increased FAK expression or enhanced activity in many human cancers, including lung (Zhou et al., 2018; Aboubakar Nana et al., 2019b), head and neck (Zhang and Sun, 2020), oral cavity (Kato et al., 2020), thyroid (Ignjatović et al., 2021), breast (Roy-Luzarraga et al., 2020), ovarian (Li et al., 2015), prostate (Goto et al., 2020), colon (Murata et al., 2008), liver (Francalanci et al., 2020), stomach (Luo et al., 2020), pancreatic (Furuyama et al., 2006), kidney (Béraud et al., 2015), skin (Najjar et al., 2020), and bone (Thanapprapasr et al., 2017; Gu and Zhou, 2018) cancer. In addition, an increased expression or activity of FAK has been reported in various cancer cell lines (Aboubakar Nana et al., 2019c). Here, we selected several representative cancers to investigate the tumor effects of FAK (Figure 2; Table 1).

**TABLE 1:** Summary of the clinical characteristics of FAK in malignant tumors.

Cancer types	Expression	Cases	Clinical characteristics	Cell phenotype	Interacting molecule	PMID
NSCLC	Upregulation	157	Not associated with survival outcome in this North American cohort	—	—	25122425
NSCLC/SCLC	Upregulation	200	Higher in SCLC	—	—	31658694
NSCLC	Upregulation	153	Associated with poorer prognosis	—	—	23143646
Lung cancer	Upregulation	—	Associated with poorer prognosis	—	—	8795582
Lung cancer	Upregulation	41	Lymph node metastasis, associated with poorer prognosis	—	—	9194028
Hepatocellular carcinoma	Upregulation	60	Associated with poorer prognosis	—	—	15246215
Hepatocellular carcinoma	Upregulation	17	Pediatric HCCs, more significant up-regulation in a cirrhotic background	—	β-Catenin	32806748
Hepatocellular carcinoma	Upregulation	97	Disease-free survival	Tumorigenicity and stemness	Wnt/β-catenin	30849480
Gastric cancer	Upregulation	32	Disease-free survival, depth of invasion, lymph node metastasis, pathological stage	—	ASAP1	32566028
Gastric cancer	Upregulation	444	Age, tumor size, distant metastasis, lymph node metastasis, venous invasion, perineural invasion	—	—	20869748
Gastric cancer	Upregulation	66	Size, disease stage, nodal status, associated with poorer prognosis	—	—	18987997
Colorectal cancer and breast cancer	Upregulation	43	-	—	—	10873094
Colorectal cancer	Upregulation	330	Associated with poorer prognosis	—	—	32739842
Colorectal cancer	Upregulation	298	Stage I, incidence of recurrence, associated with poorer prognosis	—	—	35094080
Colorectal cancer	Upregulation	45	Lymph node metastasis	Invasion	CCK2R	16998832
Colorectal cancer	Upregulation	80	—	—	—	12943621
Colorectal cancer	Upregulation	42	Liver metastases	—	—	12538472
Pancreatic invasive ductal carcinoma	Upregulation	50	Size	—	—	16425085
Urinary bladder carcinoma	Upregulation	315	Pathologic stage, disease progression, associated with poorer prognosis	—	—	31938172
Cervical cancer	Upregulation	162	Lymph node metastasis, associated with poorer prognosis	—	—	16638855
Endometrial carcinoma	Upregulation	202	Histologic grade, angiolymphatic invasion, lymph node metastasis, invasion, associated with poorer prognosis	—	—	22871469
Endometrial carcinoma	Upregulation	115	—	—	p53	15536334
Endometrial carcinoma	Upregulation	43	Age, histologic grade	—	—	21058027
Cervical Cancer	Upregulation	162	—	—	—	16638855
Cervical Cancer	Upregulation	30	Malignant transformation	—	—	12673558
Ovarian cancer	Upregulation	60	Pathological stage, histologic grade, lymph node metastasis	—	ADM	26622614
Ovarian cancer	Upregulation	79	Associated with poorer prognosis, histologic grade, lymph node metastasis	Invasion	—	29571323
Oral squamous cell carcinoma	Upregulation	70	Associated with poorer prognosis	—	—	31522363
Oral squamous cell carcinoma	Upregulation	65	—	—	p53	22790665
Head and neck squamous cell carcinoma		54	Invasion	—	—	29292531
Neuroblastoma	Upregulation	70	Advanced-stage	—	*N-MYC*	18519756
Astrocytomas	Upregulation	331	WHO grade	—	—	15221336
Osteosarcoma	Upregulation	80	Advanced-stage, recurrence	Invasion, proliferation	Akt, PDK1, BRAF	29849782
Breast cancer	Upregulation	196	Age, lymphovascular invasion, the triple-negative phenotype, associated with poorer prognosis	—	—	25326692
Breast cancer	Upregulation	162	HER2	—	HER2, Src, Akt	15743500
Breast cancer	Upregulation	102	FAK-Del26	Anti-apoptotic	—	24885534
Thyroid cancer	Upregulation	108	Size, lymph node metastasis, presence of capsular	—	—	20405349
Thyroid cancer	Upregulation	104	Lymph node metastasis	—	EGFR	29665129
Thyroid cancer	Upregulation	27	Invasion	—	—	8770310
Thyroid cancer	Upregulation	34	Pathological typing	—	—	15483349
Papillary thyroid carcinoma	Upregulation	80	Disease stage	—	—	34817652
Melanoma	Upregulation	147	P-FAKSer910 associated with better prognosis	—	—	32044881
Acute myeloid leukemia	Upregulation	60	CD34^+^	—	—	15126359
Acute myeloid leukemia	Upregulation	70	Associated with poorer prognosis	—	—	33507464
Acute myeloid leukemia	Upregulation	36	Associated with poorer prognosis	—	—	19042019
Acute myeloid leukemia	Upregulation	324	Associated with poorer prognosis	—	—	30428571
Acute myeloid leukemia	Upregulation	50	Associated with poorer prognosis	—	—	29070102

### 3.1 The molecular mechanisms of focal adhesion kinase aberrant expression and activation in tumors

#### 3.1.1 Control of focal adhesion kinase expression

Overexpression of FAK transcripts is crucial for FAK-mediated tumor cell function. The *PTK2* promoter is also activated or made more active by the transcription factors NF-κB (Corsi et al., 2006), BACH1 (Xie et al., 2022), ETV1 (Zhang et al., 2022), ETS1 (Tomar et al., 2018), NANOG (Ho et al., 2012), AGO2 (Cheng et al., 2013), and ETV4 (Li et al., 2013), which similarly increases the expression of FAK mRNA. In contrast, P53 (Cance and Golubovskaya, 2008) and ATF3 (Tian et al., 2021) limit some of the tumor’s functions by lowering the activity of the *PTK2* promoter and the number of transcripts. By directly increasing *PTK2* and *IGF1R* in hepatocellular carcinoma cells, BACH1 speeds up the development and spread of Hepatocellular carcinoma (HCC) (Xie et al., 2022). Additionally, HCC patients with ETV1/PTK2 or ETV1/c-MET co-positive hepatocellular carcinoma in two different cohorts had a worse prognosis. ETV1 can enhance HCC metastasis in HCC by upregulating PTK2 and MET (Zhang et al., 2022). ETS1, a crucial transcription factor produced by the microenvironment in ovarian cancer cells, predicts a poor prognosis and targets *PTK2* while promoting graft colonization by increasing FAK transcript levels (Tomar et al., 2018). In colon cancer cells, NANOG increases FAK expression, and FAK’s phosphorylation is a component of the signaling loop that increases NANOG activity (Ho et al., 2012). AGO2 is a component of the cellular RNA interference apparatus that is increased in hepatocellular carcinoma and stimulates FAK transcription (Cheng et al., 2013). In mice, inhibiting AGO2 lowers FAK levels while preventing tumor development and metastasis. ETV4 induced FAK expression *in vitro*, again considering its role as a transcription factor affecting *PTK2* promoter activity (Li et al., 2013). ATF3 is a downstream transcription factor of ROS, and increased levels of ATF3 can reduce the transcriptional level of FAK, reducing prostate cancer cells’ invasiveness (Tian et al., 2021). In non-coding RNA studies, CircC16orf62 was found to act as a molecular sponge for miR-138-5p and a competitive endogenous RNA for *PTK2*, which promotes the activation of the downstream AKT/mTOR pathway (Zhang et al., 2021b). Hypomethylation of the FAK promoter region was also associated with the high expression of FAK in HCC (Fan et al., 2019).

Selective splicing of mRNA (Alternative Splicing, AS) enhances the fine-tuning of protein function. By generating from an initial unique pre-messenger RNA, different protein isoforms varying in expression, subcellular localization, interactions and activities, AS represents a critical player in protein function regulation in development, physiology and disease (Kelemen et al., 2013). It was found that FAK mRNA showed three different alternative splice variants in colorectal cancer, namely FAK^0^, FAK^28^, and FAK^6^, and was associated with the invasive ability of colorectal cancer (Devaud et al., 2019). In papillary thyroid carcinoma, the number of FAK^33^ variants was elevated and positively correlated with total FAK transcripts and pY397-FAK protein levels, as well as with the advanced features of papillary thyroid carcinoma (Ignjatović et al., 2022). After comparing breast cancer tissues with normal tissues, FAK^26^ was a spliceosome expressed only in breast cancer tissues and allowed FAK proteins to acquire resistance to caspase-mediated cleavage (Yao et al., 2014). For this AS, it has been demonstrated that circRPAP2 may attenuate the selective splicing of *PTK2* by competing with *PTK2* pre-mRNA for binding to the RRM1 structural domain of SRSF1 (Yu and Fang, 2022).

#### 3.1.2 Regulation of focal adhesion kinase activity

FAK activation is mainly controlled by FAK dimerization, intramolecular inhibition of the FERM structural domain, FAK phosphorylation and other mechanisms. The most typical mechanism that promotes FAK activation involves the aggregation of integrin receptors upon cell binding to extracellular matrix (ECM) proteins, a process that involves FAK dimerization. The dimerization is formed by binding of the n-terminal FERM structural domain of FAK and is stabilized by the interaction of the FERM and c-terminal FAT structural domains. FAT binds to the basic motif on FERM that regulates coactivation and nuclear localization (Brami‐Cherrier et al., 2014). This leads to autophosphorylation of FAK at the Y397 site, binding of Src family kinases to the phosphorylation site, and mediates phosphorylation of the FAK kinase structural domain activation loop to form an activated FAK - Src complex (Lietha et al., 2007). In addition to Src, RET can also phosphorylate residues of Tyr576 and Tyr577 to activate FAK (Plaza-Menacho et al., 2011). Experiments using fluorescent biosensors have shown that when ECM binds or interacts with phosphatidylinositol lipids, the FERM structural domain undergoes conformational changes that unwind the self-inhibitory interactions (Goñi et al., 2014). Enhancing the stiffness or tension associated with cell- ECM interactions by strengthening integrin signaling has also been shown to promote FAK activation (Bauer et al., 2019), which is essential not only for mechanotransduction but also critical for tumor progression. In addition to binding partners to accelerate conformational changes in the FERM structural domain, growth factor receptors, such as MET, epidermal growth factor receptor (EGFR) and platelet-derived growth factor receptor (PDGFR), can also phosphorylate Tyr194 to relieve self-inhibition and induce FAK activation (Chen et al., 2011). In addition, Tyr397 phosphorylation is also associated with FAK activity. SHP2 is responsible for the dephosphorylation of pTyr397 and inhibits FAK activity (von Wichert et al., 2003). Phosphorylation-dependent isomerization of protein tyrosine phosphatase (PTP)-PEST promotes the interaction of PTP-PEST with FAK and the dephosphorylation of the Tyr397 site on FAK, leading to FAK inactivation (Zheng et al., 2011). SHP2 and PTP-PEST synergistically control FAK activity with Src and promote the kinetics of focal adhesion complexes, thereby facilitating cell motility (Wu et al., 2015; Chuang et al., 2021).

### 3.2 Effect of focal adhesion kinase on tumor progression

#### 3.2.1 Lung cancer

Lung cancer is a malignant tumor with high morbidity and mortality rates. As early as 1996, phosphorylated FAK was shown to be a significant component of 100–130 kDa phosphorylated proteins in lung surgery specimens and was associated with poor patient prognosis (Nishimura et al., 1996). Increased FAK phosphorylation is strongly associated with lymph node metastasis and disease-free survival in tumors (Imaizumi et al., 1997). Smoking is an important environmental factor in lung cancer, and a recent study confirmed that smoking activates the c-Src/FAK pathway (Stading et al., 2021), subsequently promoting lung carcinogenesis and progression (Zhou et al., 2019), drug resistance (Kang et al., 2013), and maintenance of KRAS-driven lung adenocarcinoma (Zhou et al., 2018). This provides ample evidence that the role of FAK in lung cancer cannot be ignored.

Lung cancer is pathologically divided into small cell lung cancer (SCLC) and non-small cell lung cancer (NSCLC). SCLC accounts for approximately 10% and has a poorer prognosis than NSCLC. Recent studies on the differences in FAK and p-FAK expression in SCLC and NSCLC have shown that the staining scores of FAK and p-FAK were significantly higher in lung cancer and SCLC tissues than in normal lung and NSCLC tissues (Aboubakar Nana et al., 2019b). There are many subtypes of NSCLC, such as lung squamous carcinoma, lung adenocarcinoma, and large cell lung cancer. FAK overexpression in NSCLC was associated with the stage as well as the adenocarcinoma subtype and positively correlated with lymph node metastasis (Ji et al., 2013). Whether there is a link between FAK expression and NSCLC prognosis is unclear and may be ethnically relevant (Ji et al., 2013; Dy et al., 2014; Aboubakar Nana et al., 2019b).

Although FAK appears to be more relevant in SCLC, most *in vitro* experiments have been conducted on NSCLC. Consistent with these pathological features, Fu et al. (2015) found that in NSCLC cells, ENO1 could enhance the proliferation, migration, invasion, epithelial-mesenchymal transition (EMT), and glycolytic capacity of tumor cells by activating the FAK/PI3K/AKT pathway. Moreover, depletion of FAK using siRNA inhibited the phosphorylation of molecules such as Src, ERK1/2, PI3K, and Akt (Fu et al., 2015). Additionally, Wang et al. (2020a) found that secretory PKM2 directly binds to integrin β1 and activates the FAK/SRC/ERK axis to promote lung cancer metastasis. Fu et al. (2020) also found that secretory OPN leads to acquired epidermal growth factor receptor tyrosine kinase inhibitor (EGFR-TKI) resistance by activating the integrin αVβ3/FAK pathway, which provides novel insights for the application of FAK inhibitors in lung cancer treatment.

### 3.2.2 Liver cancer

HCC is a prevalent disease with high morbidity and mortality rates. A study of FAK overexpression in 64 HCC tissues undergoing hepatectomy without pre-operative treatment showed that FAK expression was correlated with the clinicopathological features of HCC and was strongly upregulated in HCC compared with that in primary lesions and portal vein invasion (Itoh et al., 2004). Chen et al. (2010) reached the same conclusion and found that overexpression of FAK and its phosphorylated form in HCC tissues was associated with tumor stage, vascular invasion, and intrahepatic metastasis. The same phenomenon has been observed in human hepatoblastoma tissues (Gillory et al., 2013). In addition, several studies have demonstrated that FAK mRNA and protein expression levels are independent prognostic factors that affect disease-free survival and overall survival of patients with HCC (Fujii et al., 2004; Fan et al., 2019).

SiRNA-mediated inhibition of FAK expression in HCC cell lines revealed that the growth and apoptosis of HCC cell lines were not affected, but their adhesion and invasion abilities were reduced to different degrees (Chen et al., 2010). The FAK-ERK1/2 signaling pathway in HCC may play a vital role in reducing the stiffness of HCC stem cells and enhancing the invasive ability of HCC. These effects can be inhibited by FAK inhibitors (Sun et al., 2017; Sun et al., 2018a). Collagen is an essential component of the TME. The collagen type IV alpha1 chain (COL4A1) is known to be highly expressed in HCC and promotes the growth and metastasis of HCC by activating the FAK/Src pathway (Wang et al., 2020b). FAK is also a driver of cholangiocarcinogenesis, and *in vivo* experiments have shown that ablation of FAK significantly delayed the initiation of Akt/YAP-driven intrahepatic cholangiocarcinoma (iCCA) in mice. Additionally, growth was reduced considerably when FAK inhibitors and palbociclib (a CDK4/6 inhibitor) were administered simultaneously to mice (Song et al., 2021).

### 3.2.3 Gastric cancer

Gastric cancer is the third leading cause of cancer-related deaths worldwide (Ferlay et al., 2015). FAK is overexpressed in half of gastric cancer cases (Tani et al., 1996; Su et al., 2002; Luo et al., 2020). The same applies to the level of FAK expression in pathological specimens of patients with gastric cancer, which is positively correlated with the size, pathological stage (Luo et al., 2020), depth of infiltration, lymph node metastasis, and venous invasion of the patient’s tumor (Park et al., 2010).

Integrins also play a role in the cancer-promoting effects of FAK in gastric cancer. Annexin A6, transported in the extracellular vesicles of cancer-associated fibroblasts (CAFs), promotes drug resistance in a mouse metastatic tumor model by mediating the activation of FAK/YAP pathway in cancer cells *via* integrin β1 (Uchihara et al., 2020). In addition to drug resistance, integrin β1/FAK/YAP can mediate gastric cancer metastasis (Xiang et al., 2018). Extracellular matrix protein 1 (ECM1) mediates the activation of the FAK/SOX/HIF-1α axis by directly interacting with integrin β4 to increase metastasis and aerobic glycolysis in gastric cancer cells (Gan et al., 2018). Similarly, integrin α5β1 promotes the migration of gastric cancer cells through the FAK/ERK1 pathway (Yao et al., 2020). FAK/Akt/mTOR also seems to be the focus of research in gastric cancer, and many molecules are involved in this pathway, promoting gastric cancer growth and migration (Wu et al., 2019; Wu et al., 2021a; Qiao et al., 2021).

### 3.2.4 Endometrial carcinomas

Estrogen-dependent endometrial carcinomas express only low levels of FAK, whereas non-dependent endometrial carcinomas show FAK overexpression; p-FAK has the same expression pattern (Zhou et al., 2013a). A synergistic study on the overexpression of EZH2, FAK, and p-FAK found that all of them were positively associated with a high histological grade, type II tumors, vascular lymphatic invasion, lymph node metastasis, myometrial invasion, and cervical involvement. Contrariwise, only p-FAK overexpression was associated with omental metastasis (Zhou et al., 2013a). A study of FAK and PTEN at the pathological level also showed a positive correlation between their expression (Zhou et al., 2013b). Similarly, the expression of various molecules (such as AFP and EZH2) was found to show a synergistic increase with FAK expression in different tumor tissues (Fujii et al., 2004). This indicates, to some extent, the role of FAK in tumor development and its potential as a tumor marker. Studies on a variety of tumor tissues have revealed that the expression of many molecules such as cholecystokinin-2 receptor (Yu et al., 2006), adrenomedullin (Li et al., 2015), HER-2/neu (Lark et al., 2005), p-Src (Schmitz et al., 2005), p-Akt (Schmitz et al., 2005), PYK2 (Gutenberg et al., 2004), p120RasGAP (Hecker et al., 2004), adenosine diphosphate ribosylation factor guanylate kinase 1 (Luo et al., 2020), AFP (Fujii et al., 2004), EZH2 (Fujii et al., 2004), H3K27me3 (Francalanci et al., 2020), EGFR (Šelemetjev et al., 2018), PTEN (Zhou et al., 2013b), and pyk2 (Gutenberg et al., 2004) shows a synergistic increase with FAK expression. These molecules affect tumor development to varying degrees by interacting upstream and downstream of FAK.

### 3.2.5 Breast cancer

As one of the most common malignant diseases among women, breast cancer also displays a high degree of diversity in terms of pathological characteristics, disease progression, and response to treatment. Numerous studies have shown that FAK is downregulated in benign breast epithelium and moderately or strongly expressed in most malignant breast tissue (Weiner et al., 1993; Cance et al., 2000; Watermann et al., 2005; Almstedt et al., 2017). In particular, the high expression of FAK in early metastatic tissues suggests that it plays an important role in breast cancer metastasis (Lightfoot et al., 2004). FAK expression in breast cancer is associated with sex hormone levels. This may be related to the estrogen-related G protein-coupled receptors (Rigiracciolo et al., 2019a). High FAK expression is associated with a high histological grade, high T-stage, estrogen receptor-negative expression, progesterone receptor-negative expression, and triple-negative phenotype (Schmitz et al., 2005; Yom et al., 2011; Rigiracciolo et al., 2019a). Additionally, high FAK expression in primary foci correlates with younger patient age and lymphovascular invasion (Golubovskaya et al., 2014). Furthermore, high FAK expression is significantly and positively correlated with shorter overall survival and progression-free survival in patients with metastatic tumors (Golubovskaya et al., 2014). However, in a study of 162 lymph node-negative breast cancer tissues, FAK expression showed no prognostic significance (Schmitz et al., 2005). FAK has been suggested to play a significant role in breast cancer metastasis and affects the survival of patients with metastatic tumors.

The heterogeneity of triple-negative breast cancer with FAK-related mechanisms is possibly mediated by GPER, CTGF, and Gpx1 (Rigiracciolo et al., 2019b; Lee et al., 2020; Kim et al., 2021). Extracellular CTGF directly binds integrin αvβ3 and activates the FAK/Src/NF-κB p65 signaling axis, leading to the upregulation of Glut3 transcription, through which the glycolytic and migratory capacities of breast cancer cells are enhanced (Kim et al., 2021). Gpx1, a redox protective factor for FAK kinase, prevents kinase inactivation *via* H_2_O_2_, whereas Gpx1 deletion downregulates FAK/c-Src activation, thus preventing the spread and metastasis of tumor cells (Lee et al., 2020). Likewise, the role of FAK in the TME of breast cancer is an important research direction (Wu et al., 2020; Wang et al., 2021). Analysis of CAFs from knockout mice revealed that miR-16 and miR-148a help mediate FAK activity to enhance tumor cell activity and metastasis (Wu et al., 2020). In co-cultures of breast cancer cells and monocytes, breast cancer cells secrete CSF1 and induce monocytes to express and release CXCL7, which in turn acts on cancer cells to promote FAK activation, MMP13 expression, migration, and invasion. In a xenograft mouse model, administration of the CXCL7 antibody significantly reduced the abundance of M2 macrophages in the TME and reduced tumor growth and distant metastasis (Wang et al., 2021).

## 4 Mechanism by which focal adhesion kinase regulates tumor progression

**FIGURE 3:**
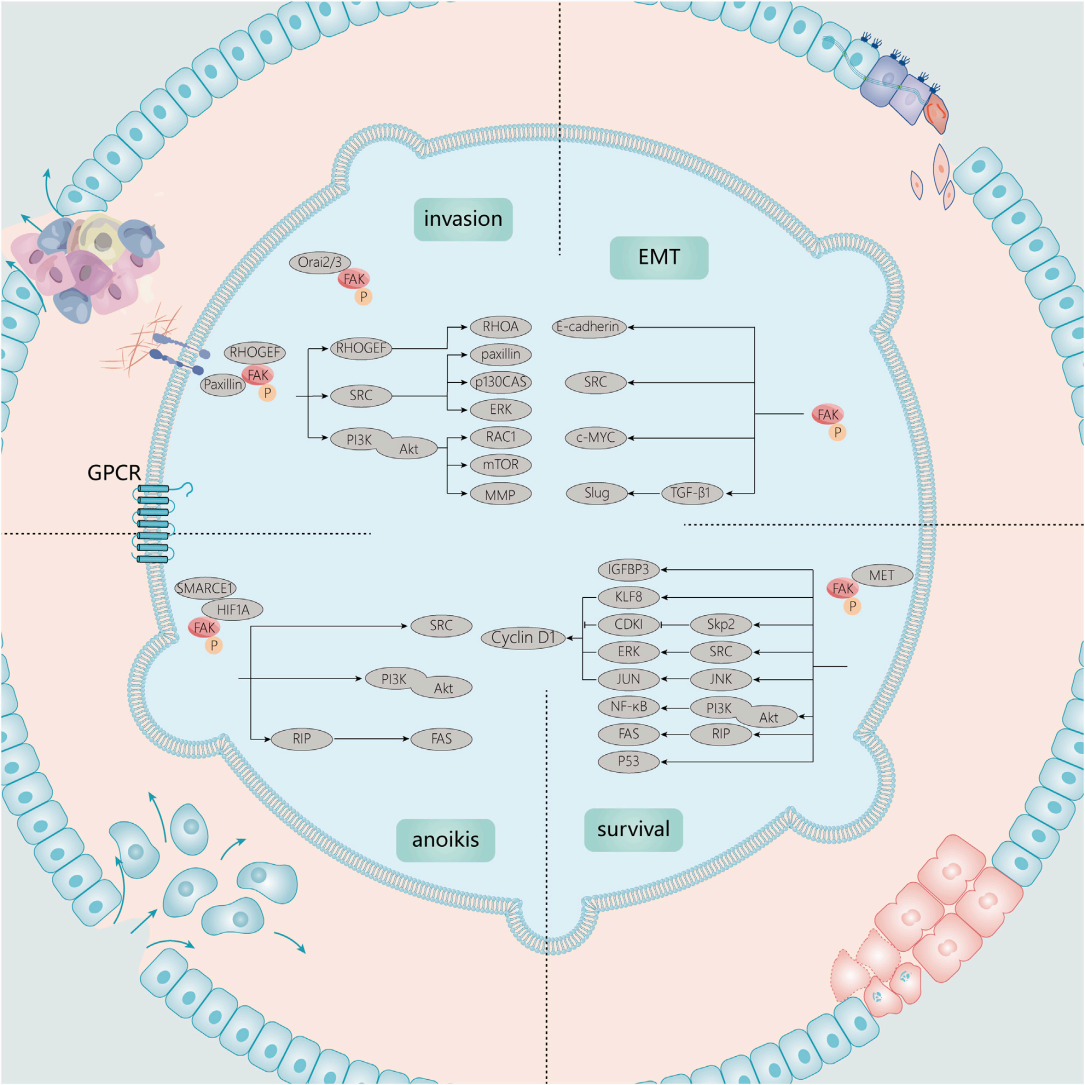
Signaling molecules associated with FAK in tumor growth and invasion. Tumor development and metastasis are complex processes. In growth signaling, FAK is closely linked to anoikis, cell cycle, and apoptotic processes. In EMT and invasion, FAK can also function through associated Src, PI3K/Akt, and other signaling pathways.

Tumor development and metastasis are complex processes that involve tumor cell shedding, invasion, migration, vascularity, and proliferation in distal parts of the body. Signaling pathways promote tumor progression and growth by regulating cell adhesion, invasion, and migration. Numerous studies on the signaling pathways between FAK and several types of cancers have revealed the biological mechanisms by which FAK promotes cancer development. This also corroborates the link between FAK overexpression and its molecular role at the pathological level (Figure 3).

### 4.1 Focal adhesion kinase and tumor survival signaling

The disorderly proliferation of malignant tumors is the major pathway of tumor progression. It is influenced by a variety of factors both inside and outside the cell. In addition, the ability of tumors to resist death, including apoptosis and anoikis, is a key aspect of tumor survival and progression.

#### 4.1.1 Cell cycle

The role of FAK in cell cycle progression has been reported previously. The gene encoding cyclin D1, a key regulator of G1/S phase progression, is the major target of FAK action in cell cycle control. Zhao et al. (1998) and Zhao et al. (2001) found that FAK could regulate cyclin D1 gene expression mediated by the ERK1/2 pathway at the EtsB-binding site (Njei et al., 2015). Expression of the autophosphorylation site-mutated FAK molecule (FAK-397F) in glioblastoma cells leads to exit from the G1 phase by decreasing the expression of cyclinD1 and E and enhancing the expression of p27 (Kip1) and p21 (Waf1) (Ding et al., 2005). In particular, in a mouse model, Marta et al. found that intranuclear FAK regulation, which is dependent on IGFBP3 transcription, regulates squamous cell carcinoma cell cycle progression and tumor growth *in vivo* and that FAK interacts with many RUNX1 regulatory proteins (Canel et al., 2017). Moreover, Zhang et al. (2019) found that MET/FAK signaling enables CDK4/6 non-dependent CDK2 activation, which leads to cell cycle delivery. Furthermore, they found that the inhibition of CDK4/6 and MET/FAK can synergistically alter the fate of tumor cells.

#### 4.1.2 Apoptosis

FAK is associated with apoptosis in cancer cells. First, FAK inhibition can lead to the loss of adhesion and apoptosis of tumor cells, which has been confirmed at an early stage (Xu et al., 1996; Xu et al., 2000). Sonoda et al. (2000) demonstrated that FAK induces IAPs by activating the PI3K/Akt pathway along with NF-κB. This ultimately inhibits apoptosis by inhibiting the caspase-3 cascade. RIP, a major component of the death receptor complex, mediates apoptosis by interacting with Fas and tumor necrosis factor receptor 1 by binding to junctional proteins. The pro-apoptotic signal provided by RIP is inhibited by its binding to FAK (Kurenova et al., 2004). In addition, the FERM structural domain of nuclear FAK interacts with the N-terminal structural domain of wild-type p53 and MDM-2 to promote the degradation of p53, thereby preventing apoptosis (Lim et al., 2008; Golubovskaya and Cance, 2011).

#### 4.1.3 Anti-anoikis

One reason for the crucial role of FAK in promoting tumor proliferation is that FAK can promote cell survival in suspension, also known as anoikis apoptosis resistance, first identified by Frisch et al. (1996). In the death receptor-mediated mechanism of anoikis, the dissociation of FAK and receptor-interacting protein (RIP) leads to the binding of RIP to FAS. This forms a death-inducing signaling complex (DISC) that activates caspase-3. Indeed, activation of the FAK/Src complex is focused on the upregulation of signaling cascades (including PI3K-Akt, ERK1/2, and other mitogen-activated protein kinases) which maintain cell survival by promoting the resistance of isolated cells to “anoikis”. In addition, the combination of FAK and RIP enhances anoikis resistance by inhibiting the binding of RIP to Fas and the formation of the death signaling complex, which allows cells to escape anoikis.

### 4.2 Focal adhesion kinase, epithelial-mesenchymal transition and invasion

Tumor cell invasion into the surrounding microenvironment is a critical step in cancer progression, allowing cancer cells to metastasize to secondary locations. This requires a shift to a motor phenotype by altering focal adhesion and cytoskeletal dynamics as well as altering matrix metalloproteinase (MMP) expression or activating to promote extracellular matrix (ECM) invasion (Weiss et al., 2022).

FAK mediates cell invasion and metastasis by promoting EMT (Canel et al., 2013; Frisch et al., 2013), in which E-cadherin plays a pivotal role as FAK mediates changes in E-cadherin expression (Avizienyte et al., 2002a; Canel et al., 2010; Serrels et al., 2011; Gayrard et al., 2018). Furthermore, SRC-FAK-dependent actomyosin remodeling relaxes E-cadherin without causing β-linked protein dissociation (Gayrard et al., 2018). FAK phosphorylation is required for Src-induced E-cadherin downregulation in colon cancer cells (Avizienyte et al., 2002b), and inhibition of FAK activity reduces Src-mediated cell invasion and blocks metastasis of FAK drug-targeted invasion and metastasis (Hauck et al., 2002). In addition, the knockdown of KIF26A increases the binding of c-MYC to the FAK promoter region and decreases the expression of E-cadherin, consequently promoting EMT (Ma et al., 2021). In parallel to E-cadherin-mediated EMT, TGF-β1-induced Slug expression also modulates EMT and promotes cell migration in human squamous cell carcinoma cells; this effect can be inhibited by FAK inhibitors (Saito et al., 2013). Accordingly, FAK plays a significant role in EMT, invasion, and metastasis. In contrast, the downstream molecular mechanisms of FAK-regulated EMT with E-cadherin-mediated cell-cell adhesion or integrin-ECM-mediated adhesion and their interactions and roles in metastasis remain to be investigated.

Invasion-associated cellular activities depend on branching networks of signal transduction pathways, including the activation of trimeric G proteins, phosphoinositide 3-kinase (PI3K), Src, signal transducer and activator of transcription, and the Rab, Rac, and Rho family of small GTPases. The heterotrimeric G protein, Gαq, activates FAK. This subsequently regulates YAP through tyrosine phosphorylation of MOB1 and inhibits core Hippo signaling (Feng et al., 2019). G-protein-coupled estrogen receptor (GPER) signaling triggers phosphorylation of Y397-FAK and an increase in adherent patches (FAs) in TNBC cells, and FAK inhibition prevents the invasion of TNBC cells upon GPER activation (Rigiracciolo et al., 2019a). Numerous reports show that FAK enhances tumor invasion through PI3K/AKT (Fu et al., 2015; Wu et al., 2019) and Src (Dong et al., 2021) signaling. In studies of melanoma invasion, STK11 was found to inhibit the invasion process of cutaneous melanoma through signal transducer and activator of transcription 3/5 and FAK repression (Dzung et al., 2022). In addition, the Rab (Choe et al., 2018; Xu et al., 2021), Rac (Acevedo-Díaz et al., 2019), and Rho (Tornin et al., 2018) families of small GTPases with FAK have been reported to affect tumor invasion in a number of ways. Some metastasis-related enzymes also play a role through the FAK signaling pathway, such as euchromatic histone methyltransferase 2 (G9a) (Sun et al., 2021), MMP-2 (Kwon et al., 2021), and PKCθ (Chadelle et al., 2022). Thus, FAK plays a vital role in the process of tumor invasion through its interaction with a range of invasion-associated molecules.

Recent studies have shown a strong relationship between calcium levels and FAK, which may also contribute to the upregulation of FAK expression and affect tumor aggressiveness. Calcium release-activated calcium modulator 2 (Orai2) is primarily upregulated during lymph node metastasis in gastric cancer. It enhances gastric cancer cell metastasis by inducing FAK-mediated MAPK/ERK activation and promotes the dissociation of focal adhesions at the posterior margin of cells (Wu et al., 2021b). Studies on Orai3 calcium channels have revealed that Orai3 alters cell adhesion capacity in two ways: 1) by reducing calpain activity, cell adhesion, and migration in a calcium-dependent manner and 2) *via* interaction with FAK to regulate the actin cytoskeleton (Chamlali et al., 2021), which is the main driver of cell adhesion and mechanosensing in a Ca^2+^-independent manner.

### 4.3 Focal adhesion kinase and tumor microenvironment

**FIGURE 4:**
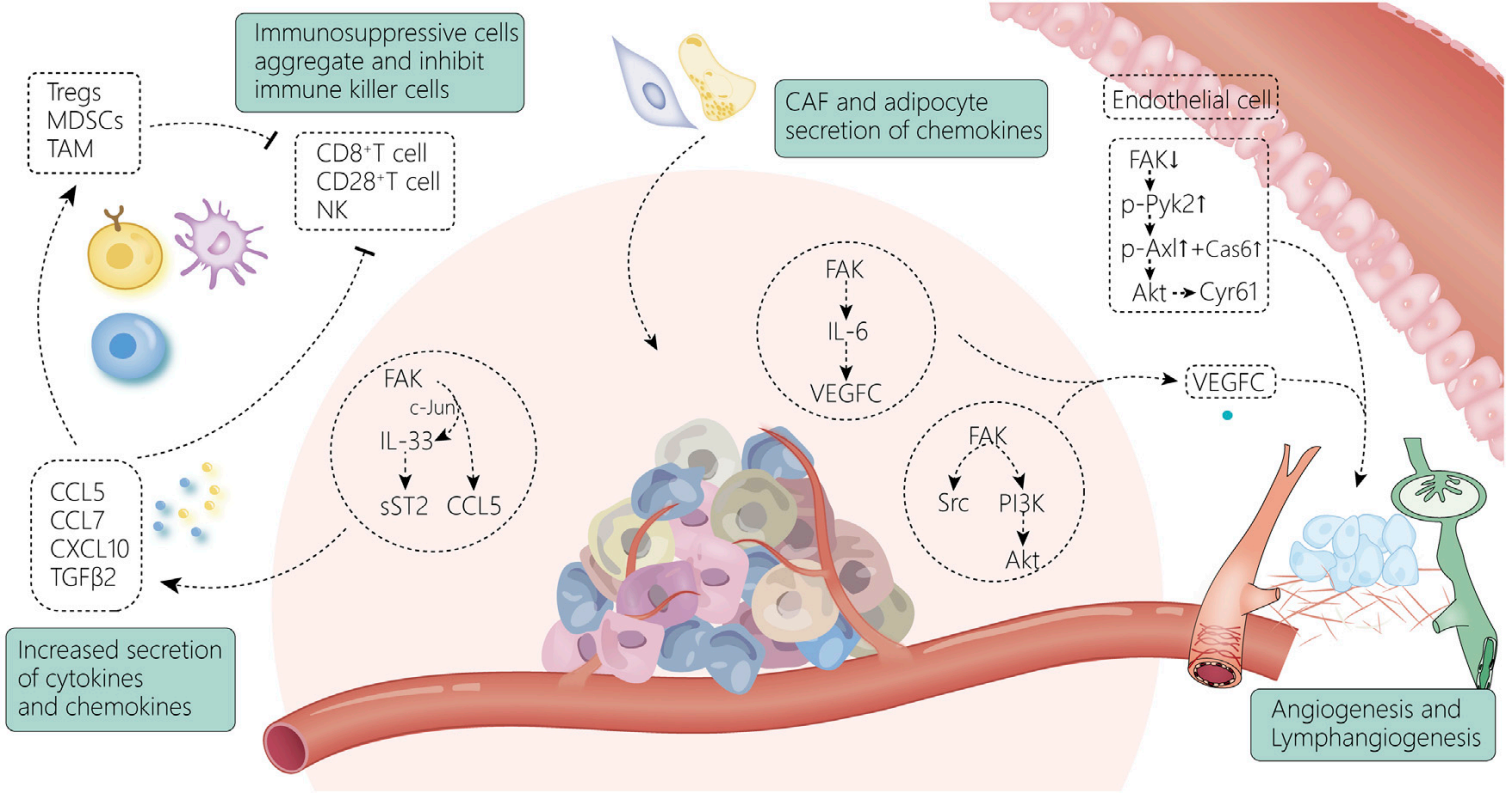
FAK and the tumor microenvironment are intricately linked. The increase in chemokines secreted by tumor cells through FAK-related pathways induces the accumulation of immunosuppressive cells and suppresses immune killer cells, allowing tumor cells to gradually escape from the immune system (Huang et al., 2016a); meanwhile, FAK activates the VEGF-C-secreted signaling pathway within the tumor tissue, leading to enhanced vascular regeneration and lymphatic vessel regeneration around the tumor. Endothelial cells (Pedrosa et al., 2019), fibroblasts (Demircioglu et al., 2020), and adipocytes (Blücher et al., 2020) surrounding the tumor can also contribute to the shaping of the TME by activating FAK-related pathways.

The TME is composed of cellular components (endothelial cells, immune cells, stromal cells, and fibroblasts) and non-cellular components (ECM, cytokines/chemokines, growth factors, and hormones) surrounding the tumor. FAK is known to play a vital role in promoting TME remodeling, which includes several processes such as angiogenesis, immune cell recruitment, and ECM (Figure 4).

#### 4.3.1 Cytokines and immune cells

FAK expression drives the establishment of an immunosuppressive TME by increasing the expression of various chemokines. It has been found that nuclear FAK increases the expression of homing signals (Huehn and Hamann, 2005; Ondondo et al., 2013), such as CCL5, CCL7, CXCL10, and TGFβ2, which are chemokines and cytokines associated with the recruitment of regulatory T cells (Tregs) (Serrels et al., 2015). This suggests that FAK activation in cancer cells plays a critical role in regulating the tumor immune landscape. FAK also enhances the expression of IL-33 (Griffith et al., 2021), and the FAK-IL-33 complex can increase the transcription of chemokine genes by interacting with CCL5 transcriptional regulators. It can also enhance the suppressive activity of Treg cells by interacting with ST2L on the surface of immune cells, thereby promoting tumor growth (Schiering et al., 2014). Alternatively, it activates the cytotoxic function of CD8^+^ T cells, resulting in improved antitumor immunity (Yang et al., 2011). FAK depletion results in the regression of CD80-expressing tumors by increasing the number of CD28^+^ T cells within the TME (Canel et al., 2020). LysM-Cre was used to knock out FAK in mononuclear phagocytes in an MMTV-polyoma middle T murine model of breast cancer, and knockout myeloid cells were found to show faster tumor growth. Increased tumor size was associated with a decrease in the number of natural killer cells, suggesting that FAK expression in myeloid cells correlates with the recruitment or survival of natural killer cells in the TME (Llewellyn et al., 2018).

#### 4.3.2 Angiogenesis

FAK has been shown to play a key role in tumor angiogenesis in multiple *in vivo* mouse models (Tavora et al., 2010; Kostourou et al., 2013). FAK in endothelial cells initiates angiogenesis, and FAK deletion reduces VEGF- and bFGF-induced angiogenesis (Tavora et al., 2010), which may be achieved through the FAK/Src/PI3K(P55)/Akt axis (Pedrosa et al., 2019). FAK affects angiogenesis and is mainly associated with Tyr397 and Tyr861 (Kostourou et al., 2013). Endothelial cell-specific expression of the FAK Y397F mutant reduces tumor angiogenesis (Pedrosa et al., 2019), where FAK affects VEGFR2 transcription through its kinase activity (Sun et al., 2018b; Shiau et al., 2021). This has also been demonstrated in recent studies, where phosphorylated Try397-FAK was found to be an important part of angiogenesis promotion in experiments in which protrudin (Arora et al., 2022) and HAX1 (You et al., 2022) affected angiogenesis. Try397-FAK can affect angiogenesis *via* ERG (D'Amico et al., 2022). In a subcutaneous Lewis lung cancer tumor model, only mice with pericyte-specific FAK-Y861F mutation showed reduced angiogenesis and tumor growth. This is associated with a notable increase in vascular degeneration (Lees et al., 2021). In addition, the detection of secretion and protein expression of FAK-Y861F pericytes revealed that cytokines and proteins promote tumor cell apoptosis and increased secretion (Lees et al., 2021). Therefore, pericyte FAK-Y861F plays a role in controlling tumor growth (Lees et al., 2021), and pericyte FAK deficiency increases tumor growth and angiogenesis (Lechertier et al., 2020a). Interestingly, when studying the specific mechanism by which FAK phosphorylation at Tyr397 and Tyr861 regulates tumor angiogenesis, it was found that *FAK* ^*Y397F/Y397F*^ *and FAK* ^*Y861F/Y861F*^ mice had different end-stage tumor vascular responses. This may be due to the enhanced p190Rhogef/p130Cas dependent signal of FAK-Y861F rather than FAK-Y397F (Pedrosa et al., 2019). Furthermore, pericyte FAK deletion enhances Gas6-stimulated phosphorylation of the receptor tyrosine kinase Axl and upregulates Cyr61, while pericyte-derived Cyr61 indicates that tumor cells upregulate the expression of the pro-angiogenic/tumorigenic transmembrane receptor tissue factor (Lechertier et al., 2020b). In addition to being a vascular signal, endothelial FAK is a regulatory site for tumor chemoradiotherapy sensitivity (Roy-Luzarraga and Hodivala-Dilke, 2016). FAK also affects ECM by promoting vascular permeability (Lee et al., 2010; Chen et al., 2012), thereby increasing the probability of tumor metastasis (Jean et al., 2014).

#### 4.3.3 Lymphangiogenesis

Lymphopenia and immunocytotoxicity are also associated with metastasis (Mlecnik et al., 2016). FAK reduces lymphocyte toxicity and affects lymphatic vessel formation (Morita et al., 2015). Among the known lymphangiogenic factors, vascular endothelial growth factor-C (VEGF-C) is the best characterized and recognized as a major regulator of lymphangiogenesis. It reshapes the lymphatic microenvironment by regulating the production of chemokines in lymphatic endothelial cells (Chen et al., 2019). FAK affects VEGF-C production *via* various signaling pathways. For example, FAK inhibition can reduce IL-6-induced VEGF-C expression and VEGF-C promoter luciferase activity (Huang et al., 2016a). Leptin-induced VEGF-C is mediated by the FAK/PI3K/Akt signaling pathway and negatively regulates the expression of microRNA-27b (Yang et al., 2016). The expression level of Nrp2 in tumor-associated lymphatic endothelial cells in colorectal cancer is significantly correlated with tumor lymphatic density. Nrp2 promotes tumor lymphangiogenesis through the integrin α9β1/FAK/Erk pathway rather than the VEGF-C/VEGFR3 signaling pathway (Ou et al., 2015).

#### 4.3.4 Extracellular matrix remodeling

Based on FAK signaling, the metabolic relationship between the ECM and the tumor is mutual. The absence of FAK in CAFs leads to enhanced glycolysis in malignant cells because FAK deletion in CAFs increases the production of chemokines CCL6 and CCL12. This in turn activates protein kinase A through CCR1/CCR2 in cancer cells (Demircioglu et al., 2020). At the same time, adipose tissue in obesity can also induce the activation of tumor FAK signaling by secreting chemokines or fatty acids and change tumor invasiveness and lipid metabolism (Blücher et al., 2020). Desmosplasia is a characteristic of most solid tumors in which PI3K plays a vital role, affecting tumor development. PI3K activation occurs when increased matrix stiffness is triggered through integrin-mediated FAK and its downstream pathway (Kallergi et al., 2007; Provenzano et al., 2008; Tung et al., 2015). The regulation of PIP3 by PI3K and the subsequent activation of Akt and mTOR are the means of remodeling the tumor environment. Through this medium, desmosplasia and increased ECM deposition affect cell metabolism, promoting cell proliferation and survival (Wozniak et al., 2003) as well as carcinogenic transformation and tumor metastasis (Levental et al., 2009). It is also the main cause of acquired chemoresistance (Darvishi et al., 2022). Therefore, FAK plays a significant role in physical construction of the TME.

### 4.4 Focal adhesion kinase and metabolic reprogramming

It has become apparent that high levels of FAK can orchestrate tumor progression by promoting metabolic reprogramming (Zhang et al., 2016). However, the specific mechanisms remain unclear.

#### 4.4.1 Glucose metabolism

After blocking FAK with siRNA and inhibitors, glucose uptake and glycolysis in glioblastoma multiforme cells were inhibited, but mitochondrial function was significantly enhanced (Che et al., 2021). In addition, fat-selective loss of FAK leads to impaired glucose tolerance and insulin sensitivity (Luk et al., 2017). Growth factors, such as insulin/IGF-1 and anchorage, are the primary extracellular cues that stimulate cell proliferation. FAK interactions with IGF1R (Kasprzak, 2021) and integrins (Che et al., 2021) transmit these growth signals by activating effectors, such as PI3K/Akt, promoting glucose consumption to fuel rapid growth of tumor cells. The N-terminal FERM structural domain of FAK binds directly to the IGF1R (Stanicka et al., 2018), leading to the activation of PI3K/Akt (Godoy-Parejo et al., 2019) and YAP (Rigiracciolo et al., 2020) signaling. Inhibition of the FAK-IGF1R interaction by small molecules induces apoptosis and inhibits tumor growth (Lehman et al., 2021). Impaired non-dependent biological functions of IGF1R kinase lead to a decrease in intracellular glucose levels, resulting in decreased cancer cell viability (Wang et al., 2022). Likewise, integrins are among the reinforcing factors in the Warburg effect of tumors (Yousefi et al., 2021). Studies have shown that FAK is a downstream effector of integrin αV/β3 and regulates the metabolic changes in glioblastoma cells to glycolysis (Che et al., 2021). CD81 can interact with integrins αV/β1 and αV/β5 to form a complex that mediates irisin-induced FAK signal transduction, and subsequently regulates the growth and energy balance of beige fat progenitor cells (Oguri et al., 2020). Twist, a key regulator of EMT, enhances aerobic glycolysis by activating β1-integrin/FAK/PI3K/AKT/mTOR and inhibiting P53 signaling (Yang et al., 2015). ECM1 significantly increased the uptake of 18^F^-deoxyglucose by xenografts, and further studies have found that ECM1 interacts with integrin β4 and induces the expression of the transcription factor SOX2 through the integrin β4/FAK/glycogen synthase kinase 3β/HIF-1α pathway. This changes the gene expression of EMT factors and glucose metabolism-related enzymes (Gan et al., 2018).

In addition, CTGF promotes aerobic glycolysis *via* the FAK/Src/NF-κB p65/Glut3 pathway (Kim et al., 2021). Hexokinase 2 (HK2) is highly expressed in ascites and metastases in patients with ovarian cancer. It is the first key enzyme to be involved in glucose metabolism. HK2 overexpression can regulate lactate production through the expression of MMP9/Nanog/Sox9 mediated by the FAK/ERK1/2 signaling pathway and participates in ovarian cancer metastasis and stem cell regulation (Siu et al., 2019).

#### 4.4.2 Lipid metabolism

As the key regulator of *de novo* lipid synthesis, fatty acid synthase (FASN) is highly expressed in many tumors. Inhibition of FASN reduces the activity of p-FAK, indicating that FAK may contribute to changes in the invasive phenotype of tumor cells caused by metabolic reprogramming (Jafari et al., 2019). Additionally, inhibition of critical lipogenic enzymes ACLY and FAS results in the reduction of FAK, Akt, and paxillin activity and cell viability (Zaytseva et al., 2012).

#### 4.4.3 Amino acid metabolism

FAK expression is related to glutamine metabolism, which may mediate changes in glutamine metabolism through the PI3K/Akt pathway, thus playing a role in cell autophagy, stress, and growth (Zhang and Hochwald, 2014). FAK stimulates PI3K/Akt signaling, whereas PI3K/Akt activation increases the levels of glutamine and its synthetase (Van Der Vos et al., 2012). The YAP/TAZ pathway plays an important role in amino acid metabolic reprogramming (Jeon et al., 2022), and FAK/Src signaling has been shown to mediate the activation of YAP/TAZ signaling in tumor cells (Totaro et al., 2018; Ma et al., 2020). However, there are still questions regarding the specific mechanisms of FAK in amino acid metabolic reprogramming, such as whether it affects the expression of key enzymes and whether there are key signaling pathways.

**FIGURE 5:**
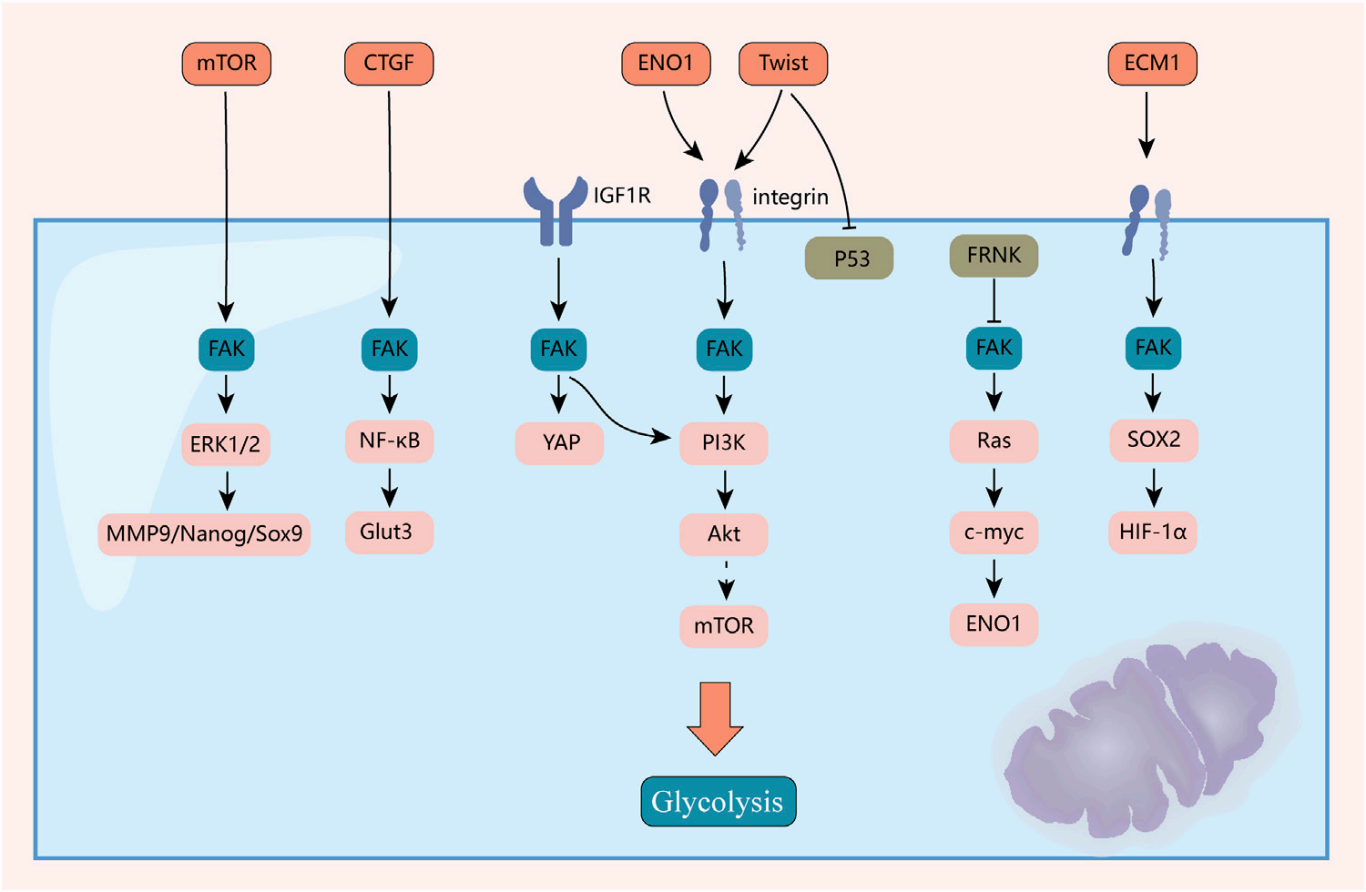
Mechanisms of FAK-mediated reprogramming of tumor glycolytic metabolism. FAK plays an important role in reprogramming the metabolism of the three major nutrients. This figure highlights the mechanisms by which it plays a role in reprogramming sugar metabolism.

Whether FAK has a core pathway and function through the metabolism of the three nutrients remains to be investigated, which is an important part of future research on the Warburg effect (Figure 5).

### 4.5 Focal adhesion kinase and tumor stemness

Cancer stem cells (CSCs) are important for clonal growth and metastasis of solid tumors. FAK may contribute to CSC activity in diverse types of tumors (Sun et al., 2018a; Diaz Osterman et al., 2019). In a histological study of liver cancer, we found that FAK expression in liver cancer patients was positively correlated with the expression of liver cancer stem cell markers (Fan et al., 2019).

Type I collagen increases the initiation potential, self-renewal ability, and frequency of CSCs in pancreatic ductal adenocarcinoma by activating FAK (Begum et al., 2017). In colon cancer cells, knockdown of transmembrane heparan sulfate proteoglycan syndecan-1 significantly enhances the stem cell phenotype of SDC-1-deficient cells by enhancing the FAK-Wnt signaling axis (Kumar Katakam et al., 2021). In malignant pleural mesothelioma, significant decreases in stem cell markers can be caused by inhibition of PFKFB3, and, thus, the disruption of the FAK-Stat3-SOX2 nexus (Sarkar Bhattacharya et al., 2022). In studies related to the transformation of normal stem cells into tumor stem cells (CSCs) without genetic manipulation, fibroblast growth factor 2 (FGF2) was found to induce normal stem cells to acquire stemness expression of tumor stem cells and initiate cancer; this process was found to be associated with integrin/FAK/PI3K/AKT signaling pathway activation (Sheta et al., 2021). In oral squamous cell carcinoma (OSCC), KRT17 regulates stemness marker levels *via* the integrin/FAK/Src/ERK/β-catenin pathway (Jang et al., 2022). In addition to its contribution to the maintenance of tumor stemness, the effect of FAK on the stemness of embryonic stem cells has been identified by a wider range of researchers (Baumann, 2021; Hur et al., 2021).

## 5 Potential for focal adhesion kinase applications in tumor biomarker and therapy

FAK small-molecule inhibitors can be divided into two major groups: 1) inhibitors that target the enzymatic or kinase-dependent functions of FAK, such as inhibitors that target the structural domain of the ATP-binding site and variant inhibitors that target other sites of FAK but still block kinase activity, and 2) inhibitors that target the scaffold function of FAK (Golubovskaya, 2014). The application of FAK inhibitors can directly and synergistically enhance the therapeutic and killing effects on tumor cells and restore the sensitivity of a few drug-resistant tumor cells.

As FAK mediates resistance to treatment, the application of FAK inhibitors can restore the sensitivity of some tumor cells after chemoresistance. In high-grade serous ovarian cancer models *in vivo*, Y397-FAK phosphorylation increased upon sublethal cisplatin treatment of platinum-resistant tumors (Diaz Osterman et al., 2019). Since platinum-induced cell stress can activate FAK, it has been suggested that FAK activation may function to permit acquired platinum tumor resistance (Diaz Osterman et al., 2019). In addition, FAK inhibition allows resistant tumors to regain cisplatin sensitivity (Mohanty et al., 2020). Cancer patients treated with EGFR inhibitors often develop resistance to treatment. Some evidence suggests that EGFR-TKI resistance works through an integrin-mediated pathway (Seguin et al., 2014), and FAK is involved in the increase in resistance of cancer cells to EGFR-TKI (Solanki et al., 2018). The combination of erlotinib and FAK inhibitors has been shown to be effective in reducing the survival of EGFR-TKI-resistant NSCLC cells (Murakami et al., 2017). In addition, according to Grace et al., during epithelial cell migration, the complex formed by EGFR and FAK has a common downstream Ezrin, and FAK and/or Ezrin could be targeted and/or used in combination with EGFR to overcome the resistance of cancer cells to EGFR-TKI in the future. Reversal of EMT and repolarization of tumor-associated macrophages (TAMs) using simvastatin targeting the role of FAK in lipid metabolism can treat drug-resistant cancers (Jin et al., 2019). The YAP pathway leading to tumor drug resistance is now a comparatively clear mechanism (Nguyen and Yi, 2019). FAK is required for Y357-FAK phosphorylation, and both play a vital role in intrahepatic cholangiocarcinoma (ICCA) development through the FAK/Akt/YAP pathway. ICCA growth was significantly reduced when treated with both FAK inhibitor and CDK4/6 inhibitor palboclib in both *in vivo* and *in vitro* experiments (Song et al., 2021). FAK inhibitors synergize with KRAS G12C inhibitors to treat different cancers; this process is also accomplished through the FAK-YAP signaling pathway (Zhang et al., 2021c).

FAK inhibitors can synergistically increase the sensitivity of various cancers to chemotherapeutic agents because they not only reduce FAK expression (Le Large et al., 2021; Tong et al., 2022) but also inhibit numerous signaling pathways associated with FAK. In a study on the effect of tyroservatide (YSV) on lung cancer cell metastasis, YSV was found to inhibit the adhesion and invasion of human lung cancer cells and had a therapeutic effect on lung cancer metastasis. YSV significantly inhibited the phosphorylation of FAK Tyr397 and FAK Tyr576/577 in highly metastatic human lung cancer cells (Huang et al., 2016b). *In vivo* experiments have shown that endothelial cell-specific FAK deletion sensitizes tumor cells to DNA damage treatment, thereby reducing tumor growth in mice (Newport et al., 2022). In addition, treatment with adriamycin may alter vascular, secretory signaling associated with improved chemosensitivity of acute tumor cells in FAK^−/−^ mice compared with that in wild-type mice (Newport et al., 2022). FAK inhibitors can inhibit tumor progression by altering epigenetic forms. TAE226, in combination with SOR, effectively reduced HCC growth, both *in vitro* and *in vivo*. TAE226-mediated FAK deletion and SOR-promoted MAPK downregulation led to a decrease in HDAC1/2 expression in the nucleus, which in turn increased histone H3 lysine 27 acetylation (H3K27ac). This inhibited histone H3 lysine 27 trimethylation (H3K27me3) and suppressed tumor progression through altered epigenetic forms (Romito et al., 2022).

**FIGURE 6:**
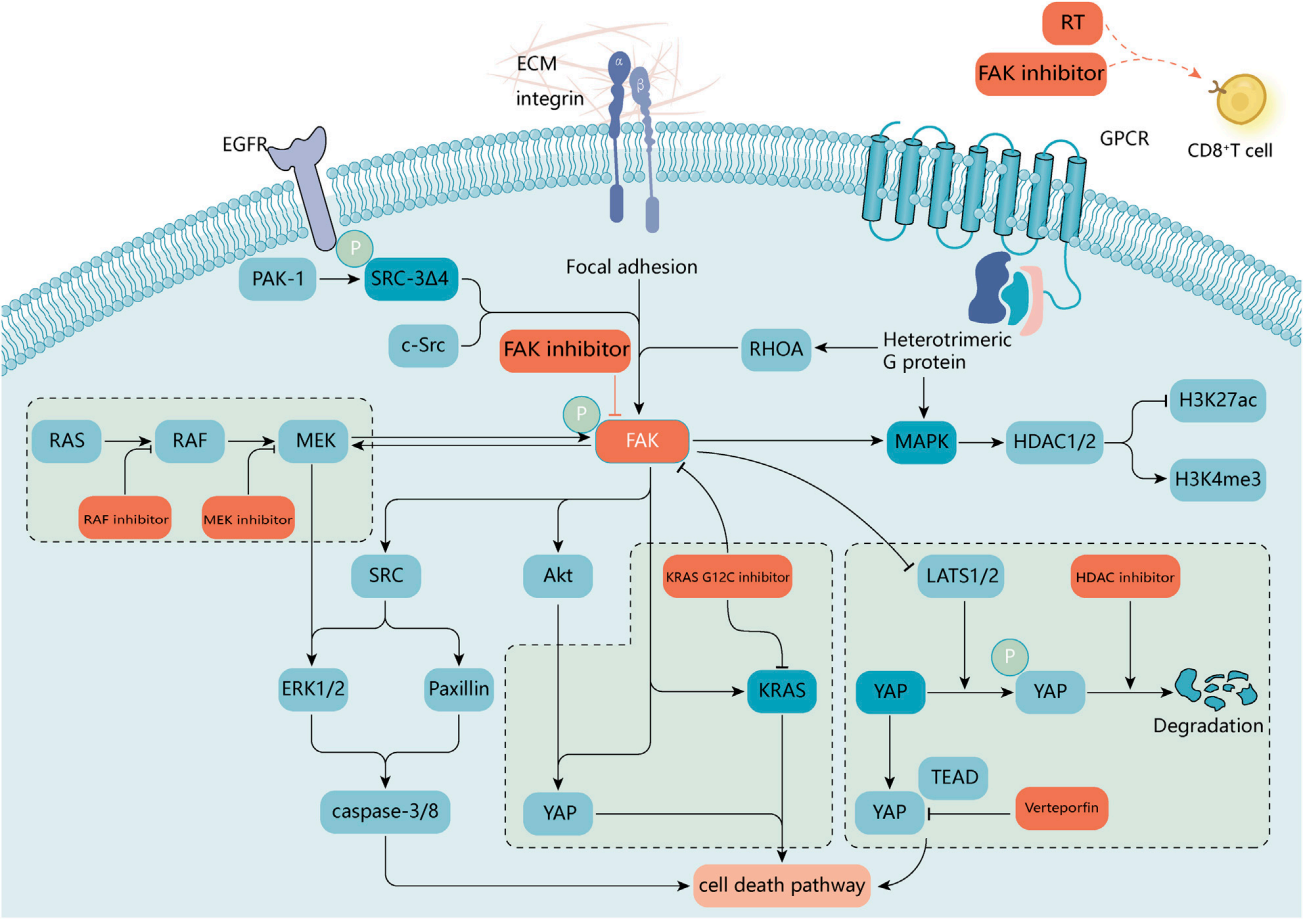
A molecular target for combination therapy with FAK inhibitors. FAK supports a variety of oncogenic processes and is beneficial in combination with a variety of available antitumor agents. In RAS-mutated or RAF-mutated cancer cells, blocking the RAS-RAF-MEK pathway with RAF or MEK inhibitors activates FAK and promotes cell survival by reactivating ERK signaling. Activated FAK in diffuse gastric cancer and uveal melanoma attenuates the negative regulation of the transcriptional activator YAP by large tumor suppressor 1 and 2 (LATS1/2). FAK activity can promote nuclear translocation of YAP, and combinations of FAK inhibitors with inhibitors of YAP expression [e.g., histone deacetylase (HDAC) inhibitors] or transcriptional activity may be required to enhance inhibition of oncogenic YAP signaling (Hicks‐Berthet and Varelas, 2017; Song et al., 2021). Inhibition of RHOA or FAK selectively induces mutant KRAS cell death in non-small cell lung cancer studies (Konstantinidou et al., 2013). In breast cancer sRc-3Δ4, a splice isoform of the oncogene was found to be a signaling adapter linking EGFR and FAK and promoting EGF-induced phosphorylation of FAK and c-Src (Long et al., 2010). FAK inhibitors also play a role in epigenetics (Romito et al., 2022), and radiation treatment in combination with FAK inhibitors affects the immune microenvironment surrounding the tumor (Osipov et al., 2021). The small GTPase, RAS homolog family member A (RHOA), regulates the actin cytoskeleton. ECM, extracellular matrix; GPCR, G protein-coupled receptor.

Inhibition of FAK renders tumors more sensitive to radiotherapy (RT) (Eke et al., 2012; Zhang et al., 2021d). In 2002, Kasahara et al. first reported that FAK overexpression significantly enhanced radiation resistance in human leukemia cells. The results of this study showed that FAK overexpression inhibited the caspase-8 expression and caspase-3 activation, thereby exerting resistance to ionizing radiation (IR)-induced apoptosis. This process has since been found to be mediated through various signaling pathways, such as paxillin, Akt1, JNK, and ERK1/2 (Hehlgans et al., 2012; Ou et al., 2012). This is not only related to DNA damage repair, EMT-related protein expression, and cell cycle arrest but may also be related to the immune microenvironment (Skinner et al., 2016a; Skinner et al., 2016b; Hou et al., 2016; Tang et al., 2016). CD8^+^ T-cell infiltration was significantly enhanced after treatment with FAK inhibitor combined with RT. Additionally, granulocyte infiltration was significantly reduced, and macrophage and T-cell infiltration was significantly increased in the FAK inhibitor combined with radiotherapy group compared with that in the radiotherapy alone group (Osipov et al., 2021) (Figure 6; Tables 2, 3).

**TABLE 2:** Summary of preclinical studies with FAK inhibitors.

Inhibitor	Molecular targets	Cancer types	PMID
BI-853520 (IN10018)	FAK	Prostate cancer; breast cancer	29472531; 30237500
GSK2256098	FAK	Pancreatic cancer; ovarian cancer	25486573; 27064283
NVP-TAC544	FAK	N/A	18391070
PF-431396	FAK/PYK2	Pancreatic cancer	19244237
PF-573228	FAK	Pleural mesothelioma; lung cancer	29303405; 17395594
TAE226	FAK/IGF-IR	Breast cancer; ovarian carcinoma; hepatocellular carcinoma	17849451; 17431114; 34784956
VS-4718	FAK/PYK2	Breast cancer/ovarian cancer; pancreatic cancers	27376576; 20234191
VS-6062	FAK/PYK2	Gliomas; pancreatic cancer; colon cancer; lung cancer; prostate cancer; breast cancer	18339875; 18339875; 22454420
VS-6063	FAK/PYK2	Ovarian cancer; Hepatocellular carcinoma	24062525; 35154476
C4	FAK-VEGFR3 interaction	Breast cancer	19610651
R2	FAK-p53 interaction	Colorectal cancer	23841915
Y11	FAK	Colon cancer and breast cancer	22402131
Y15	FAK	Breast cancer; lung cancer	18989950; 27336608

**TABLE 3:** Summary of clinical trials with FAK inhibitors.

Name	Tumor	Target	Status/phase	Trial identifiers
Defactinib (VS-6063)	NSCLC	FAK	Completed	NCT01951690
Defactinib (VS-6063)	Malignant pleural mesothelioma	FAK	Terminated	NCT02004028
Defactinib (VS-6063)	Solid cancer	FAK	Completed	NCT01943292
Defactinib (VS-6063) VS-6766	Ovarian cancer	FAK MEK	Phase 2	NCT04625270
Defactinib (VS-6063) VS-6766	NSCLC	FAK MEK	Phase 2	NCT04620330
Defactinib (VS-6063) VS-6766	NCT04720417	FAK MEK	Phase 2	NCT04720417
Defactinib (VS-6063) VS-6766	Cervical cancer high grade Serous ovarian cancer	FAK MEK	Phase 2	NCT05512208
Defactinib (VS-6063) Pembrolizumab	Pancreatic ductal adenocarcinoma	FAK PD-1	Phase 2	NCT03727880
Defactinib (VS-6063) Pembrolizumab	Pancreatic cancer NSCLC	FAK PD-1	Phase 2	NCT02758587
Defactinib (VS-6063) Paclitaxel	Ovarian cancer	FAK Tubulin	Completed	NCT01778803
Defactinib (VS-6063) Pembrolizumab Gemcitabine	Advanced solid tumors; Pancreatic cancer	FAK PD-1 DNA	Phase 1	NCT02546531
Defactinib (VS-6063) radiation therapy	Pancreatic cancer	FAK DNA	Phase 2	NCT04331041
GSK2256098	Solid cancer	FAK	Completed	NCT01138033
GSK2256098	Solid cancer	FAK	Completed	NCT00996671
GSK2256098 Trametinib	Advanced solid cancer	FAK MEK	Completed	NCT01938443
VS-4718	Metastatic cancer	FAK	Terminated	NCT01849744
VS-4718 Nab-paclitaxel Gemcitabine	Pancreatic cancer	FAK Tubulin DNA	Terminated	NCT02651727
PF-04554878	Solid cancer	FAK	Completed	NCT00787033

The high expression of FAK in a wide range of tumors, as illustrated in Part II of this paper, suggests its potential as a diagnostic marker. When combined with clinical data, FAK expression levels are found to correlate with prognostic levels in tumors such as liver cancer, gastric cancer, colorectal cancer, bladder cancer, OSCC, breast cancer, thyroid cancer, AML, and melanoma; therefore, FAK has essential qualities as a prognostic marker.

## 6 Conclusion

In this study, we first analyzed the molecular pathology of FAK expression in various tumor types. We found that it was not only overexpressed in tumors but also correlated with clinical features, such as tumor stage and prognosis of cancer patients. We then described how FAK overexpression exerts regulatory effects at the molecular level in tumor cells and their surroundings. This process participates in many cancer-related processes, such as tumor invasion, EMT, construction of the TME, metabolic reprogramming, and maintenance of tumor stemness. The role of FAK in clinical applications is also summarized. FAK inhibitors combined with other established chemotherapeutic agents can reduce the rate of treatment resistance and further enhance the tumor-killing capacity.

As mentioned earlier, future research on FAK could be combined with the clinical characteristics of patients with tumors to specifically explain how the function of FAK hair differs in pre-, mid-, and late-stage tumor patients. Research on FAK inhibitors is important as it could potentially lead to treating patients with tumors in the future. The scientific justification for the clinical application of FAK needs to be refined. In addition, the potential of FAK for therapeutic and diagnostic purposes is promising and can further oncology research.
